# Unravelling site-specific breast cancer metastasis: a microRNA expression profiling study

**DOI:** 10.18632/oncotarget.13623

**Published:** 2016-11-25

**Authors:** Willemijne A.M.E. Schrijver, Paul J. van Diest, Dutch Distant Breast Cancer Metastases Consortium, Cathy B Moelans

**Affiliations:** ^1^ Department of Pathology, University Medical Center Utrecht, Utrecht, The Netherlands

**Keywords:** microRNA, breast cancer, metastasis, expression profiling, site-specificity

## Abstract

Distant metastasis is still the main cause of death from breast cancer. MicroRNAs (miRs) are important regulators of many physiological and pathological processes, including metastasis. Molecular breast cancer subtypes are known to show a site-specific pattern of metastases formation. In this study, we set out to determine the underlying molecular mechanisms of site-specific breast cancer metastasis by microRNA expression profiling.

To identify a miR signature for metastatic breast carcinoma that could predict metastatic localization, we compared global miR expression in 23 primary breast cancer specimens with their corresponding multiple distant metastases to ovary (n=9), skin (n=12), lung (n=10), brain (n=4) and gastrointestinal tract (n=10) by miRCURY microRNA expression arrays. For validation, we performed quantitative real-time (qRT) PCR on the discovery cohort and on an independent validation cohort of 29 primary breast cancer specimens and their matched metastases.

miR expression was highly patient specific and miR signatures in the primary tumor were largely retained in the metastases, with the exception of several differentially expressed, location specific miRs. Validation with qPCR demonstrated that hsa-miR-106b-5p was predictive for the development of lung metastases. In time, the second metastasis often showed a miR upregulation compared to the first metastasis.

This study discovered a metastatic site-specific miR and found miR expression to be highly patient specific. This may lead to novel biomarkers predicting site of distant metastases, and to adjuvant, personalized targeted therapy strategies that could prevent such metastases from becoming clinically manifest.

## INTRODUCTION

With a worldwide incidence of 1.67 million and a mortality of 522,000 patients, breast cancer is the leading cause of female cancer and the fifth cause of overall cancer death [1]. The majority of solid tumor related mortality is caused by metastatic progression [2], rendering the genetic changes and molecular mechanisms by which cancer cells acquire their metastatic ability one of the most important challenges in breast cancer research. MicroRNAs (miRs) may be involved here, as critical regulators of global mRNA expression in both physiological and pathological processes, including cancer [3].

miRs are a group of small non-coding RNAs able to regulate gene expression at the post-transcriptional level by binding to target mRNAs [4]. Dysregulation of miRs occurs in various types of cancer and is associated with tumor initiation, drug resistance, and metastasis. Therefore, therapeutic strategies based on modulating the expression levels of miRs and identifying their targets are promising approaches for cancer treatment [5].

Even though there have been several studies investigating the role of individual miRs in breast cancer metastasis, often only focussing on their presence in primary tumors [6-8], few global miR expression profiling studies have yet been performed in paired primary breast tumors and their solid distant metastases [9]. For lymph node metastases, a metastatic miR signature has already been identified comprising over- and underexpressed miRs [10, 11]. Extensive knowledge of the miRs involved in distant metastasis could lead to novel biomarkers predicting site of distant metastases and adjuvant targeted therapy strategies that could prevent such metastases from becoming clinically manifest.

Intrinsic (molecular) subtypes of breast cancer have been shown to preferentially metastasize to specific sites. E.g., while luminal ERα-positive cases prefer to seed to the bone, triple negative and HER2-driven cancer metastases often go to the brain [12]. We therefore set out to study global miR expression patterns of 23 primary breast cancer specimens and their corresponding multiple solid distant metastases on selected locations, to pinpoint changes in miR expression during progression from the primary tumor to specific distant sites.

## RESULTS

### miRs differentially expressed in primary breast cancer versus corresponding multiple distant metastases

**Figure 1:**
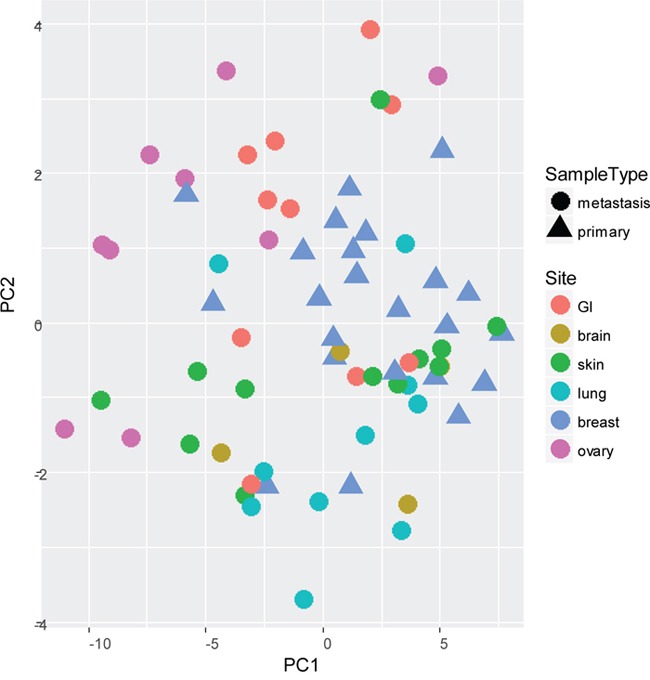
Principal Component Analysis (PCA) of most varying components (PC1 and PC2) between all primary tumors (n=23) and paired multiple distant metastases (n=46) of cohort 1, subjected to miRCURY microRNA expression array profiling

First, the samples of cohort 1 were subjected to miRCURY microRNA expression array profiling. A principal component analysis (PCA) of the samples showed only small variances between the common reference channels, indicating that the observed variances of the tissue samples were likely related to biological differences between the tumor samples and not technical variances (Supplementary Figure S2). When PCA was performed for the most varying components (PC1 and PC2), the primary tumors and some but not all of the metastases locations (GI, skin and lung) clustered only vaguely together (Figure 1). Molecular subtype and patient number seemed to be the most important clustering variables.

**Figure 2:**
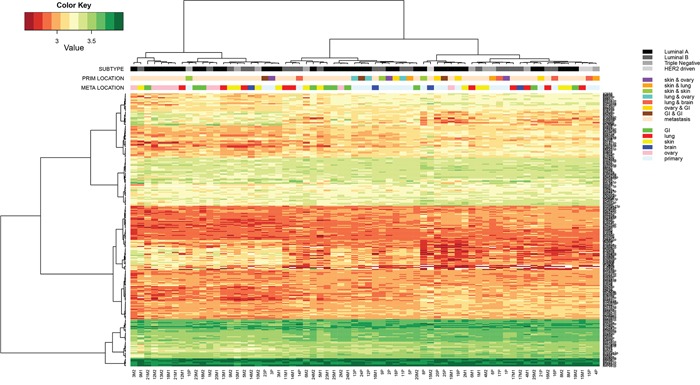
Hierarchical cluster analysis of Threshold filtered data of all primary tumors (n=23) and paired multiple distant metastases (n=46) of cohort 1, subjected to the miRCURY microRNA expression array

An unsupervised cluster analysis of all detected miRs did not readily separate the samples into groups, but some agreement in miR expression was seen in ER+ primary tumors and GI and ovarian metastases. No clear distinction was observed based on the samples being either primary tumors or distant metastases, but for 9/23 (39%) of the patients, the primary tumor and (one of) the corresponding distant metastases clustered together, indicating very similar miR expression patterns (Figure 2). For this group, the time between the primary and the metastasis was significantly shorter (p=0.021; mean 947 days; range 0-3867 days) than for the group that did not cluster together (mean 1988 days; range 0-8965). However, when individual miR expression was correlated to time between primary and metastases, no significant relation was found. For another 9/23 of the patients, the two metastases clustered roughly together, but here no significant differences were seen in time span when compared to patient samples that did not cluster.

In non-paired analyses, 48 miRs were identified that were differentially expressed between primary tumors and metastases (21 upregulated and 27 downregulated in the metastases versus the primary tumors). In paired analyses per metastasis location, 101 miRs were identified that exhibited a significantly altered expression between paired primary tumors and metastases (Supplementary Table S3). Almost no overlap was seen in differentially expressed miRs per metastatic location, only hsa-miR-3201 was dysregulated in lung and ovarian metastases. Interestingly, compared to other metastatic localizations, ovaries generally demonstrated more differentially expressed miRs (n=86 versus n=4 for skin, n=11 for lung, n=2 for GI and n=0 for brain).

### Validation of miRs differentially expressed in primary breast tumors versus corresponding distant metastasis as analyzed by quantitative PCR analysis

The following analyses were performed on cohort 1: i) primary tumors versus metastases (paired and unpaired), ii) primary tumors that disseminated to a specific site (brain, lung, GI, ovary or skin) and iii) differences between molecular subtypes. The miRs with the highest fold changes in these analyses were validated using real-time PCR. Moderate to good correlations were seen between microarray data and qPCR validation (Supplementary Table S4). Subsequently, these miRs were validated in the independent cohort 2. Only hsa-miR-16-5p was excluded from further validation due to low correlations. qPCR validation of hsa-miR-200a-3p, hsa-miR-29b-3p, hsa-miR-451a, hsa-miR-125b-5p, hsa-miR-143-3p and hsa-miR-3182 in both cohorts are shown in Supplementary Figure S3.

### Comparison between multiple metastases per patient

**Figure 3:**
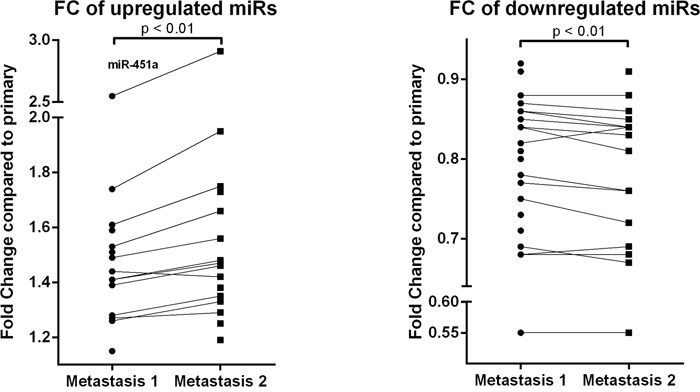
Fold change of significantly upregulated (a) and downregulated (b) miRs of both metastases (compared to the primary tumor) Results of the Threshold filtered data of the miRCURY microRNA expression array of cohort 1. miR-451a shows the highest upregulation. Wilcoxon signed-rank test was used with * p < 0.05, ** p < 0.01, *** p < 0.001

A general tendency was observed towards a higher expression in the second metastasis compared to the first. Especially in the significantly upregulated miRs, an increase in fold change was shown in the second metastasis (compared to the primary tumor) relative to the first metastasis (Figure 3). However, no significant correlation was seen between miR expression and time between primary and metastasis. As an example, the miR with the highest upregulation (miR-451a; FC M1: 2.55 and FC M2: 2.91) was plotted against timespan between primary and metastasis and metastatic location (Supplementary Figure S4). Location of metastasis and patient specific differences were the largest contributors to the differences in miR expression. Time to distant recurrence was not significantly different (p=0.637) between the metastatic locations of cohort 1 (Supplementary Figure S5).

### Expression differences in primary tumors and metastases of miRs known to have an oncogenic and tumor suppressive potential in primary breast tumors

**Figure 4:**
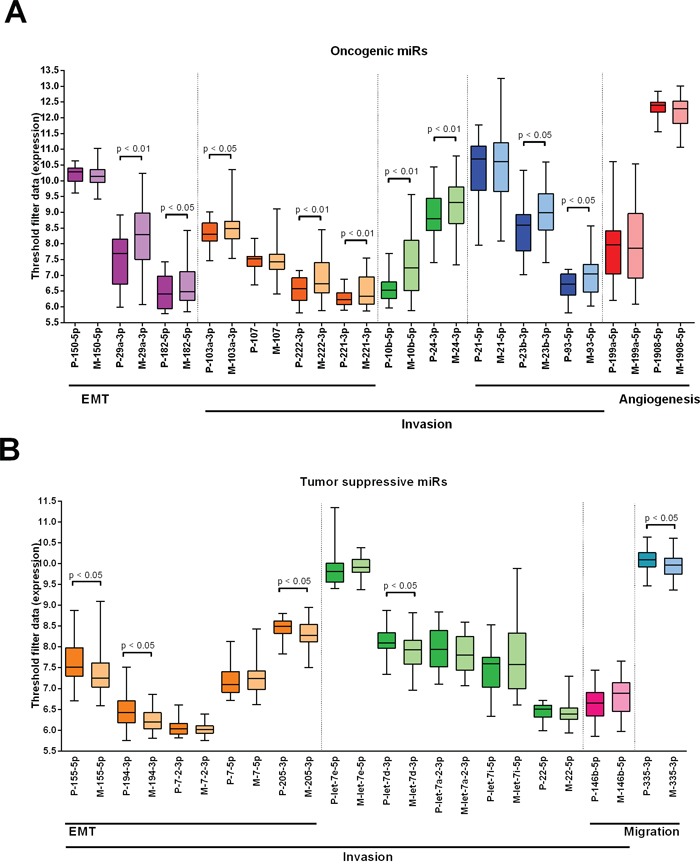
Expression differences in primary tumors and metastases of miRs known to have an oncogenic and tumor suppressive potential in primary breast tumors Data from cohort 1. **A.** Expression of 15 oncogenic miRs with a role in EMT, invasion and angiogenesis in primary tumors versus metastases. **B.** Expression of 12 tumor suppressive miRs with a role in EMT, invasion and migration in primary tumors versus metastases. Wilcoxon signed-rank test was used with * p < 0.05, ** p < 0.01, *** p < 0.001. Purple: miRs with a role in EMT, orange: miRs with a role in EMT and invasion, green: miRs with a role in invasion, blue: miRs with a role in invasion and angiogenesis, red: miRs with a role in angiogenesis, pink: miRs with a role in invasion and migration, turquoise: miRs with a role in migration.

A literature search resulted in 38 oncogenic and tumor suppressive miRs that play a role in the metastatic cascade in primary tumors [4, 8, 13] (Supplementary Table S5). Expression of these miRs was evaluated in the primary tumors compared to the metastases of cohort 1 and 26 oncogenic (n=14) and tumor suppressive (n=12) miRs were expressed in all tested samples. Of the 14 selected oncogenic miRs, 9 miRs with a role in EMT, invasion and angiogenesis were significantly upregulated in the metastases compared to the primaries (Figure 4a). Of the 12 selected tumor suppressive miRs, 5 miRs were significantly downregulated in metastases (Figure 4b).

### Prediction of site-specific metastasis by miR expression in the primary tumor

**Figure 5:**
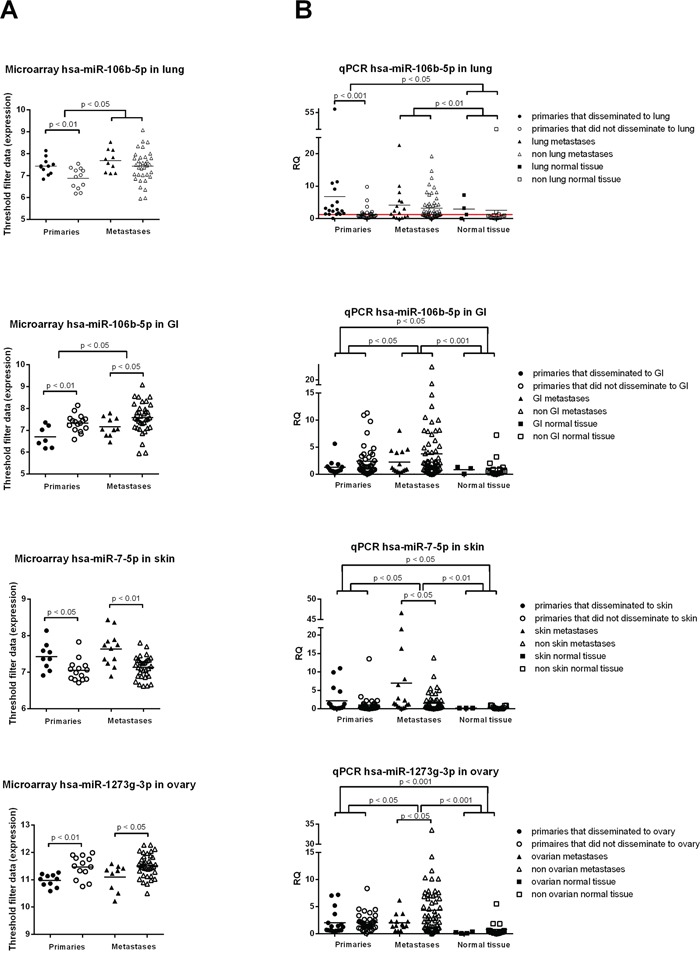
MiRs shown to be predictive in the primary tumor for metastasis location **A.** with microarray profiling of cohort 1, miR-106b-5p was an independent predictor for lung and GI metastases, miR-7-5p for skin metastases and miR-1273g-3p for ovarian metastases. **B.** qPCR validation on the same cohort plus an independent cohort revealed the same trend as in A), but only miR-106b-5p remained an independent predictor for lung metastases. Mann-Whitney U test was used with * p < 0.05, ** p < 0.01, *** p < 0.001

miRs predicting metastasis location based on expression levels in the primary tumor are listed in Supplementary Table S3. In a multivariable regression model corrected for molecular subtype, histologic type, histologic grade, tumor diameter, lymph node status, age at diagnosis and MAI, miR-106b-5p was an independent predictor of lung and GI metastases, miR-7-5p of skin metastases and miR-1273g-3p of ovarian metastases (Figure 5a). These findings were validated by qPCR in cohorts 1 and 2. Only miR-106b-5p remained an independent predictor of metastases to the lung. ROC-curve analysis showed an AUC of 0.828 (95% CI 0.701-0.955; SE 0.07; Supplementary Figure S6) with an RQ value of ≥1.208 (sensitivity 0.94; 1-specificity 0.34). Although not significant, in all three tested miRs (hsa-miR-106b-5p, hsa-miR-1273g-3p and hsa-miR-7-5p) the same expression trend was observed in the metastases. In (independent) normal tissue, a significantly lower expression was seen compared to primary tumors and metastases (Figure 5b).

To ascertain the role of hsa-miR-106b-5p, hsa-miR-1273g-3p and hsa-miR-7-5p in the metastatic process, we used miRTarBase, the experimentally validated microRNA-target interactions database [14]. The mRNA targets with strong validation evidence (obtained by reporter assay, western blot or qPCR) are listed in Supplementary Table S6. These target genes were subsequently imported in ToppGene Suite [15] to find enriched pathways. Unfortunately, for hsa-miR-1273g-3p, there are no known targets with strong evidence. Hsa-miR-106b-5p appears to have an important role in both lung and breast cancer. Furthermore, this miR is a key player in cell cycle control and regulation and the cellular response to stress. Hsa-miR-7-5p plays a role in breast cancer, melanoma and bacterial invasion of epithelial cells. Regarding metastatic properties, focal adhesion, apoptosis and angiogenesis are significantly enriched pathways.

## DISCUSSION

Systemic therapies are still largely guided by the characteristics of the primary tumor, while discordance between primary tumors and metastases are often encountered [16-19]. Molecular differences between matched primary tumors and metastatic lesions have the potential to reveal novel, potentially targetable drivers of metastatic progression. In this study we performed miR expression profiling in primary breast tumors and matched multiple metastases. We demonstrated that the expression of known ‘metastamiRs’ was generally higher in the metastases compared to the primary tumors. Also, the abundance of specific miRs in the primary tumor seemed to be metastasis location-specific, which could potentially be exploited to gain more knowledge about the metastatic cascade. Especially hsa-miR-106b-5p seems to be a predictor of lung metastases.

Several studies have examined the role of individual miRs in primary metastatic breast cancer. For example, miR-148a, miR-33a, miR-34a and miR-199a/b-3p are thought to suppress metastasis [20] and to inhibit tumor cell migration and invasion [21-23]. In contrast, miR-762 and miR-1228, amongst others, are thought to promote breast cancer cell proliferation and invasion [24] and metastasis [25]. However, little is known about the full miR profile of primary breast tumors compared to paired distant metastases. Gravgaard et al. already performed expression profiling of primary breast tumors and matched distant metastases to liver (n=5) and brain (n=9) [9]. In line with Gravgaard et al., we observed a higher miR expression similarity between primary tumors and metastases when the recurrence interval was shorter. However, a time dependent effect on the individual miR level was not found. Furthermore, they reported 97 altered miRs between primary tumors and brain metastases, while we found none, possibly due to our smaller sample size (n=4).

Baffa et al. compared 13 primary breast tumors and matched lymph node metastases and found five upregulated and six downregulated miRs [10]. Only upregulation of hsa-miR-30b and downregulation of hsa-miR-125b corresponded to our findings, suggesting that some miRs have an influence on the metastatic cascade in general, while others could correlate to location specificity. In another study that compared miR expression in primary breast tumors and lymph node metastases (n=97) a downregulation of hsa-miR-151-5p was seen in metastases, while we detected an upregulation [26]. This may be explained by the fact that our metastases were distant, with different microenvironments and progression routes playing a role. This is further supported by the fact that specific miRs can have an oncogenic potential in one cancer type and a tumor suppressive effect in the other [27].

Several gastric and colorectal cancer studies discovered the same possible pro-metastatic miRs as we did, suggesting that these miRs can influence cancer progression in general (miR-10a: [28]; miR-335: [29]; miR-143: [30]). However, there is virtually no overlap in candidate miRs across prior studies. Whether this arises from dissimilarities in tumor types, metastasis locations, patient characteristics or the use of varying techniques remains unclear. Findings with qPCR and microarray did not always correlate well [31], which was in this study overcome by selecting only miRs with high fold changes for validation.

Overall, there was a tendency for higher expression of certain oncogenic miRs in the metastases compared to the primaries. This observation is in line with Huang et al., who reported a higher expression of hsa-miR-373 in paired lymph node metastases of 11 patients [32]. In addition, they reported higher levels of hsa-miR-373 in the primary tumors that disseminated to lymph nodes compared to lymph node negative samples. We saw a similar tendency for hsa-miR-106b-5p, but this should be validated in a larger group. Also Korpal et al. found a higher expression of hsa-miR-200s in paired lung metastases, stressing its potential role in metastatic colonization [33].

Overall, only few differentially expressed miRs were found between primary tumors and matched metastases (with an exception for ovarian metastases), suggesting that miR expression is largely retained in metastases. Ferracin et al. already showed that primary tumors of different origin display a distinct miR expression profile and that metastases retain a large part of these miRs [34]. The latter was visualized by unsupervised hierarchical clustering, where primary tumors and metastases clustered together. We also observed some clustering of primaries and paired metastases, but the metastatic locations hardly clustered. This could be due to patient specific differences or to too much variation within subsites (different GI and skin locations).

Why the ovarian metastases differ so clearly from the other metastatic sites remains to be elucidated. The H&E slides were reinspected by an experienced breast pathologist, who was convinced about their origin from the breast. However, there is still a small chance that a part of these tumors are primary ovarian cancers since metastatic carcinomas may mimic primary ovarian carcinomas. Also, mucinous ovarian carcinomas are difficult to distinguish from metastatic adenocarcinomas [35]. The biggest microRNA expression differences were seen between normal tissue and tumor tissue (primaries and metastases). Our findings agree with Neerincx et al. who described that miR expression in primaries and metastases is similar, but differs largely from normal tissue [36]. Other studies frequently use ‘normal tissue’ of regions near the tumor, which may introduce bias when presumably normal tissue has already been affected. By comparing to reference material of all metastasis locations we tried to rule out tissue-specific background as much as possible. Because of limited availability, we used (unpaired) normal tissue that did not originate from the same patients, which may have introduced patient-specific background differences.

Smeets et al. developed a predictor of lymph node metastases based on miR expression profiling of the primary tumor [11]. Here, we demonstrated that high expression of hsa-miR-106b-5p in the primary tumor can predict lung metastases. A review about the influence of miR-106 in cancer showed a moderate accuracy in identifying gastric and colorectal cancer and lymphoma patients [37]. In breast cancers, miR-106b was found to be associated with a high risk of recurrence, and was mentioned as a putative plasma marker for risk assessment [38].

Certain limitations to our study include the small samples sizes per metastatic location and potential tumor heterogeneity, which may explain some of the observed differences between primary tumors and paired metastases. Furthermore, by making use of microarray technology we may have underestimated downregulated miRs, since the applied threshold prevents the detection of lowly expressed miRs.

In summary, we have shown that primary tumor miR expression patterns are largely retained in metastases, except from some location specific miRs. miR-106b-5p expression in the primary tumor seems to be an independent predictor of lung metastases. miR-7-5p, miR-1273g-3p and miR-106b-5p could be predictors for skin, ovarian and GI metastases as well, respectively, but these results require validation in a larger and independent cohort. This miR expression profiling study thereby identified possible therapeutic targets and predictive markers of site-specific metastasis. The large patient specific differences further stress the uniqueness of individual tumors and thereby the need for individualized treatment.

## MATERIALS AND METHODS

### Patients

From a series of 481 patients gathered at the department of pathology of the University Medical Center Utrecht in The Netherlands within the framework of a Dutch Cancer Society project on the genotype and phenotype of distant breast cancer metastases [16-19], we selected 25 formalin-fixed paraffin embedded (FFPE) tissue specimens of female primary breast carcinomas and per patient two corresponding distant metastases to lung (n=10), brain (n=4), skin (n=12), ovary (n=10) and gastro-intestinal sub sites (GI; n=10) (cohort 1). Per patient, the metastatic locations could be subdivided into lung-skin (n=3), lung-ovary (n=3), lung-brain (n=4), skin-ovary (n=3), skin-skin (n=3), GI-GI (n=3) and ovary-GI (n=4). Independent validation was performed in 29 matched patients (cohort 2; matched according to age at diagnosis of the primary, molecular subtype, location and time to metastasis) with single metastases to ovary, skin, lung, brain and gastro-intestinal subsites. Clinicopathological characteristics of both cohorts are shown in Table 1. To correct for tissue specific differences in miR expression, 4 independent normal tissues were selected per tumor location except brain (Supplementary Table S1). Molecular IHC-surrogate subtypes of breast tumors were assigned as follows: Luminal A-like (ER+/PR+, HER2−, Ki-67<15), luminal B-like (ER+/PR+, HER2−, Ki-67>15 or ER+/PR+, HER2+), triple negative or basal-like (ER-/PR-, HER2-) and HER2 enriched (ER-/PR-, HER2+), as before [12].

**Table 1:** Clinicopathological characteristics of 23 primary tumors with two paired metastases included for microRNA expression profiling (cohort 1: microarray profiling and qPCR validation) and a validation cohort of 29 primary tumors and single paired metastases (cohort 2: qPCR validation)

Characteristics	Subgroup	Cohort 1 (n=23)		Cohort 2 (n=29)		p
		N	%	N	%	
Number of samples	Primaries	N=23		N=29		
	Metastases	N=46		N=30		
Age at diagnosis (in years)	Range	29-72		31-88		0.151
	Mean	49		53		
Tumor diameter (in cm)	Range	0.5-4		1,5-10		0.057
	Histologic type	Ductal	N=16	69.6%	N=19		65.5%	0.814
Lobular		N=5	21.7%	N=8	27.6%			
Other		N=2	8.7%	N=2	6.9%			
Histologic grade (Bloom & Richardson)	I	N=2	8.7%	N=4	13.8%	0.745
	II	N=11	47.8%	N=13	44.8%	
	III	N=10	43.5%	N=12	41.4%	
MAI (per 2mm^2^)	Range	0-50		0-102		0.719
	Mean	15		20		
Molecular subtype	Luminal A	N=12	52.2%	N=16	55.2%	0.823
	Luminal B	N=7	30.4%	N=4	13.8%	
	Triple negative	N=3	13.1%	N=8	27.6%	
	HER2-enriched	N=1	4.3%	N=1	3.4%	
Lymph node status	+	N=10	43.5%	N=15	51.7%	0.037*
	-	N=4	17.4%	N=10	34.5%	
	Unknown	N=9	39.1%	N=4	13.8%	
Metastasis location	Lung	N=10	21.7%	N=6	20.7%	0.491
	Brain	N=4	8.7%	N=7	24.2%	
	Skin	N=12	26.2%	N=5	17.2%	
	Ovary	N=10	21.7%	N=6	20.7%	
	GI	N=10	21.7%	N=5	17.2%	
Time between primary tumor and metastasis (in days)	Range	0-8965		225-3296		0.222
	Mean	1752		1340		
- Lung	Range	467-5502		367-2480		0.828
	Mean	1615		1250		
- Brain	Range	631-1224		371-2970		0.850
	Mean	896		1379		
- Skin	Range	0-3458		605-1872		0.225
	Mean	1682		1161		
- Ovary	Range	0-8965		225-2345		0.346
	Mean	2525		1278		
- GI	Range	-1719-3944		714-3296		0.758
	Mean	1603		1687		
Metastasis subgroups	Lung-skin	N=3	13.0%			
	Lung-ovary	N=3	13.0%			
	Lung-brain	N=4	17.5%			
	Skin-ovary	N=3	13.0%			
	Skin-skin	N=3	13.0%			
	GI-GI	N=3	13.0%			
	Ovary-GI	N=4	17.5%			

* significant difference between the two cohorts.

The experiments were performed in accordance with the institutional medical ethical guidelines. The use of anonymous or coded left over material for scientific purposes is part of the standard treatment agreement with patients and therefore informed consent was not required according to Dutch law [39].

### RNA extraction

Four-μm thick sections were cut from each FFPE tissue block and stained with haematoxylin and eosin (H&E). The H&E-section was used to guide macro-dissection and to estimate tumor percentage. Only samples containing 80 per cent tumor load or higher (both primary tumor and metastases) were selected. Four 10-μm-thick slides were cut and deparaffinized in xylene. Tumor areas were macro-dissected using a scalpel and areas with necrosis, dense lymphocytic infiltrates, and pre-invasive lesions were intentionally avoided. RNA extraction was carried out with the miRNeasy FFPE kit (Qiagen) according to the manufacturer's instructions and samples were eluted in 25μL RNAse free water. Total RNA concentration was measured spectrophotometrically (Nanodrop ND-1000, Thermo Scientific Wilmington, DE, USA). Only samples with a concentration of >50 ng/μL and a total amount of 500ng RNA were included for microarray analysis, resulting in 23 matched primary tumor and multiple metastases pairs.

### miR array profiling

All experiments were conducted at Exiqon Services, Denmark. The quality of the total RNA was verified by an Agilent 2100 Bioanalyzer profile. 400 ng total RNA from sample and reference was labeled with Hy3™ and Hy5™ fluorescent label, respectively, using the miRCURY LNA™ microRNA Hi-Power Labeling Kit, Hy3™/Hy5™ (Exiqon, Denmark) following the procedure described by the manufacturer. The Hy3™-labeled samples and a Hy5™-labeled reference RNA sample were mixed pair-wise and hybridized to the miRCURY LNA™ microRNA Array 7th Gen (Exiqon, Denmark), which contains capture probes targeting all miRs for human, mouse or rat registered in the miRBASE 18.0. Hybridization was performed according to the miRCURY LNA™ microRNA Array Instruction manual using a Tecan HS4800™ hybridization station (Tecan, Austria). After hybridization the microarray slides were scanned and stored in an ozone free environment (ozone level below 2.0 ppb) in order to prevent potential bleaching of the fluorescent dyes. The miRCURY LNA™ microRNA Array slides were scanned using the Agilent G2565BA Microarray Scanner System (Agilent Technologies, Inc., USA) and the image analysis was carried out using the ImaGene 9.0 software (BioDiscovery, Inc., USA). The quantified signals were background corrected (Normexp with offset value 10, see [40]) and normalized using quantile normalization method, to enable good between-slide normalization to minimize the intensity-dependent differences between the samples.

### qPCR validation

Reverse transcription was performed with the Universal cDNA Synthesis Kit II (miRCURY LNA™ microRNA PCR, Polyadenylation and cDNA synthesis, Exiqon, Denmark). qPCR was performed in duplicate on the ViiA™ 7 Real-Time PCR System (Applied Biosystems) with the ExiLENT SYBR® Green master mix (Exiqon) and ROX as a passive reference. Each run included non-template controls and a calibrator sample. An appropriate endogenous control miR was selected based on the threshold-filtered data from the array profiling. MiRs were included in the analysis if i) they were found in all samples, ii) they had a probe signal of at least 7 in the lowest sample and iii) they had average signal of at least 7.5 across all samples. This data set was run through the NormFinder algorithm [41] to get a stability value for the expression of each miR in the data set. Filtering the most stable hits by assay availability resulted in hsa-miR-483-3p as the best candidate. For the qPCR validation figures, the test and validation cohorts were combined.

### Prediction of metastasis location by assessing specific miRs in the primary tumor

Mann-Whitney U test analyses were performed to compare miR expression in primaries that disseminated to a specific site versus primaries that disseminated to all other tested sites (the rest). For these analyses we also searched the anonymised medical histories of these patients, to find out if they also had metastases in the selected organs (brain, GI, lung, skin, ovary) of which no tumor material was present (Supplementary Table S2). With this information we corrected for selection bias.

To ascertain the role of the candidate location-specific miRs in the metastatic cascade, we used miRTarBase, the experimentally validated microRNA-target interactions database [14]. The mRNA targets with strong validation evidence (obtained by reporter assay, western blot or qPCR) were imported in ToppGene Suite [15] for pathway enrichment analysis.

### Statistical analyses

The Threshold filter data obtained by miR array profiling of almost 2098 miRs was manually checked and non-human miRs were excluded. miRs with no signal or a signal <7 in >25% of samples were excluded as well. Roughly 700 miRs were expressed above background in every sample (Supplementary Figure S1).

Unsupervised hierarchical clustering of Threshold filtered data was performed using non-parametric Spearman correlation with R (version 3.2.5). Non-paired analyses on patient differences and clinicopathological characteristics were computed using the Kruskal-Wallis and Mann-Whitney U test. Paired analyses between primary tumors and metastases were done using the Wilcoxon signed-rank test. P-values below 0.05 were considered significant. Thereafter, correction for multiple comparisons was performed by the Benjamini Hochberg procedure. Univariable and multivariable relationships were tested by logistic regression (method: Forward LR) with 95% confidence intervals (CI). All statistical calculations were done with IBM SPSS Statistics 21 and visualized with GraphPad Prism 6 and R.

We thank Stichting PALGA for the national query for cases.

This publication was realized with support of ‘Stichting Vrienden UMC Utrecht’ and a Sister's Hope. We would like to thank Annelisa Cornel for helping with some qPCR assays and Antonio Sorrentino for advice and contact with Exiqon Services, Vedbæk, Denmark.

## SUPPLEMENTARY MATERIALS FIGURES AND TABLES










