# Supplementary material for: Unravelling site-specific breast cancer metastasis: a microRNA expression profiling study
Source: Oncotarget. 2016 Nov 25;8(2):3111–23. doi: 10.18632/oncotarget.13623 (PMC5356868; doi:10.18632/oncotarget.13623)
Supplement: Supplementary file 1 [file oncotarget-08-3111-s001.pdf]

Unravelling site-specific breast cancer metastasis: a microRNA expression profiling study

SUPPLEMENTARY FIGURES AND TABLES

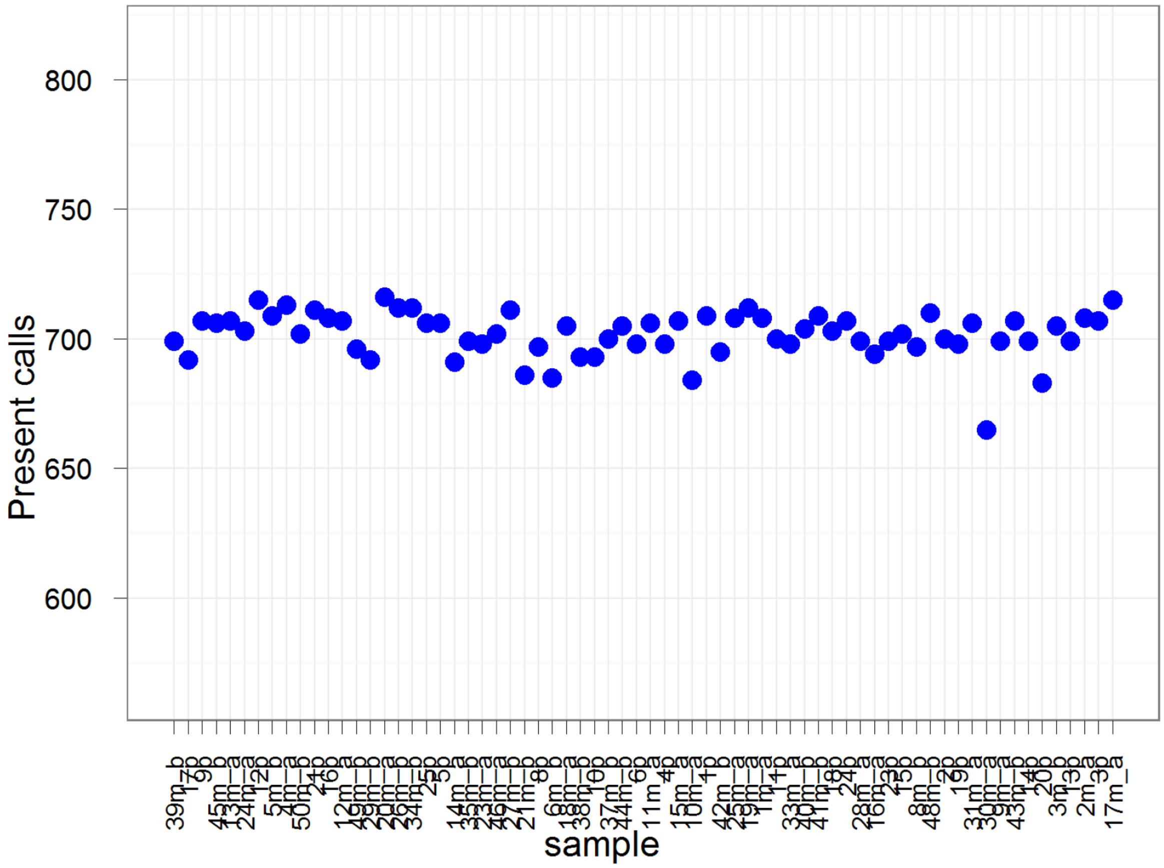

Supplementary Figure S1: Plot illustrating the number of microRNAs detectable in the microarray above background threshold (1.2 times the 25th percentile of the overall signal intensity of the slide) for each sample of cohort 1(out of a total of 2098 possible microRNAs).

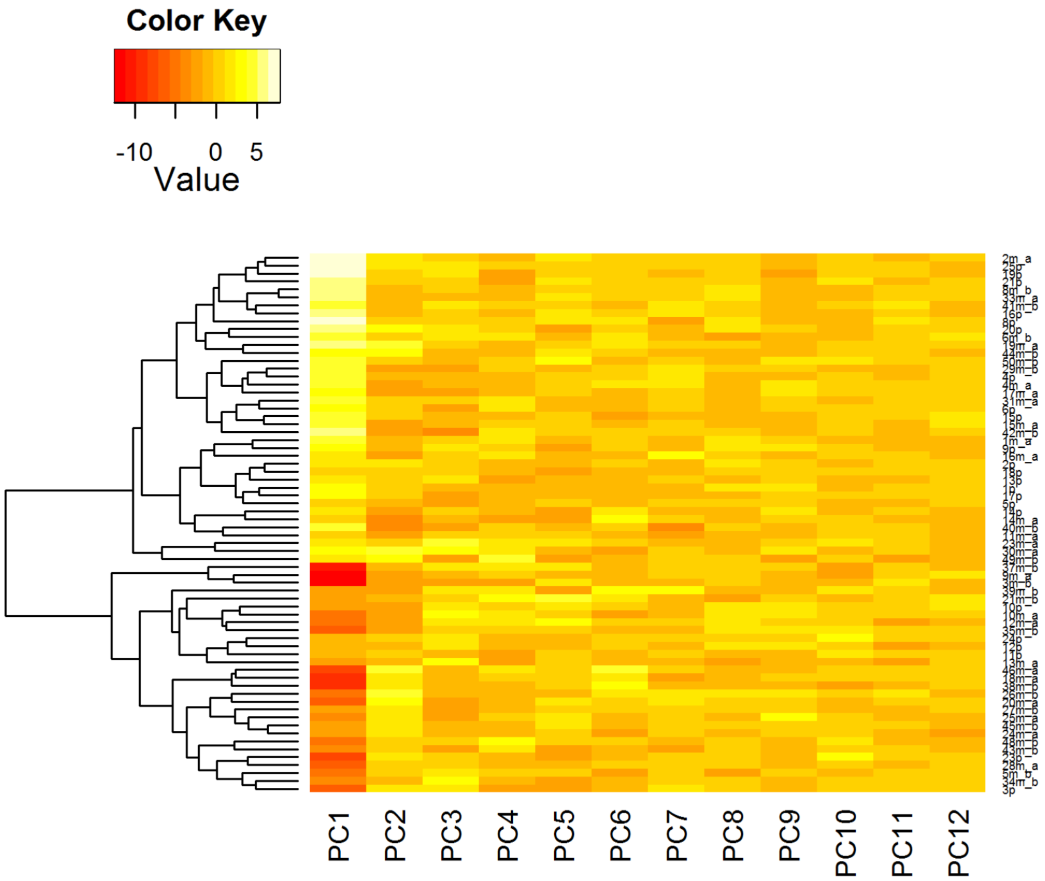

Supplementary Figure S2: Matrix PCA plot of the common reference channels of the microRNAs detectable with the microarray of cohort 1. Analyses were performed on all tumor samples. The observed variances of the tissue samples were likely related to biological differences between the tumor samples and not technical variances, because most variation was seen in PC1 and PC2.

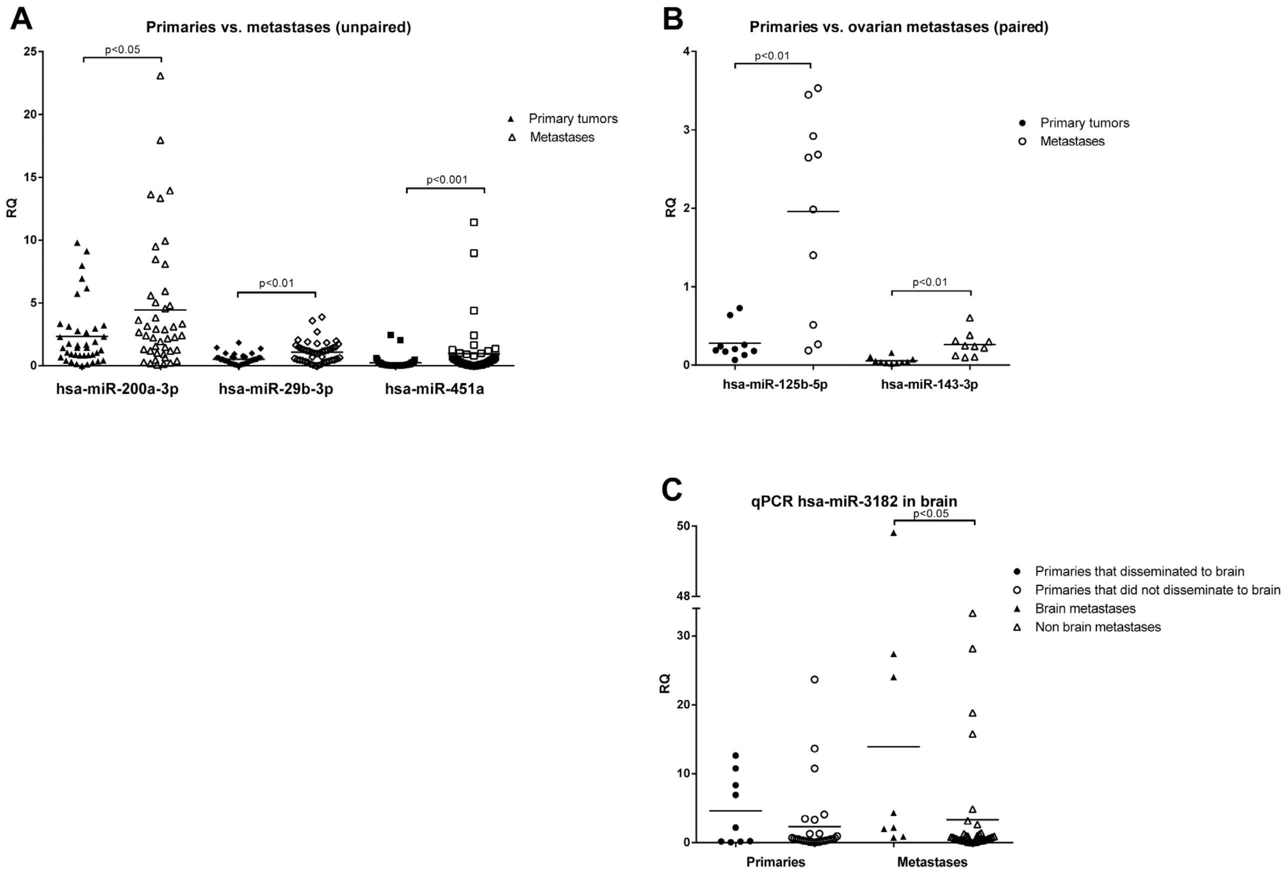

Supplementary Figure S3: Validation with qPCR of significantly deregulated miRs in primary tumors versus metastases (paired and unpaired) of cohort 1 and 2. * $p < 0.05$, ** $p < 0.01$, *** $p < 0.001$

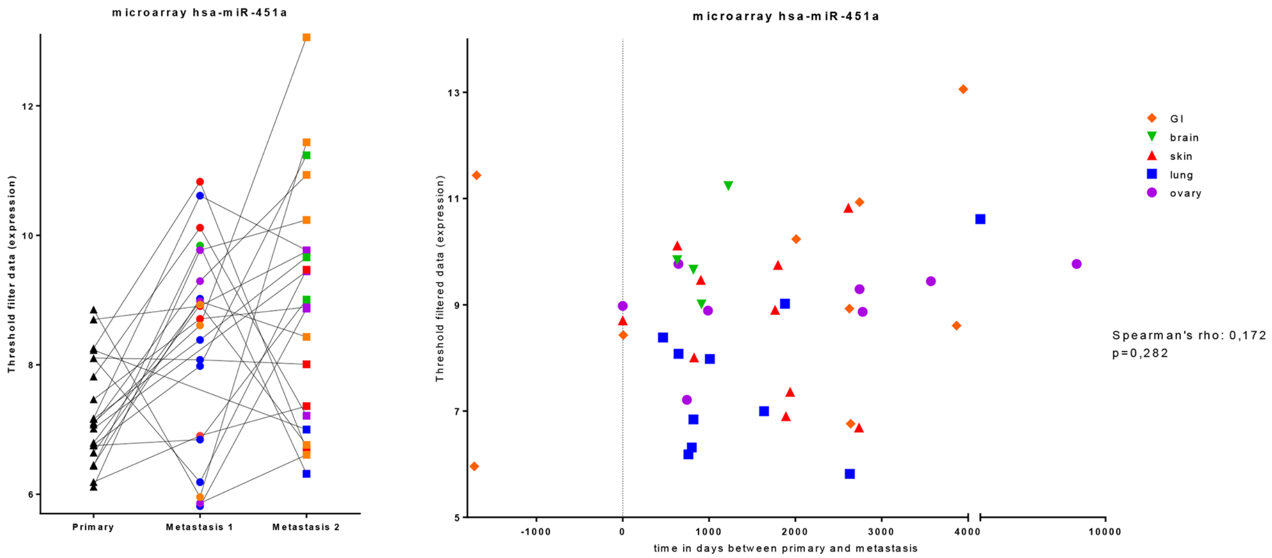

Supplementary Figure S4: Threshold filtered data of the miRCURY microRNA expression array of miR-451a (highest fold change of metastases compared to the primary tumor) in cohort 1. A. Expression of all metastases 1 and 2 compared to their paired primary tumor. **B.** expression of all metastases compared to the time interval between the primary tumor. No significant correlation was seen.

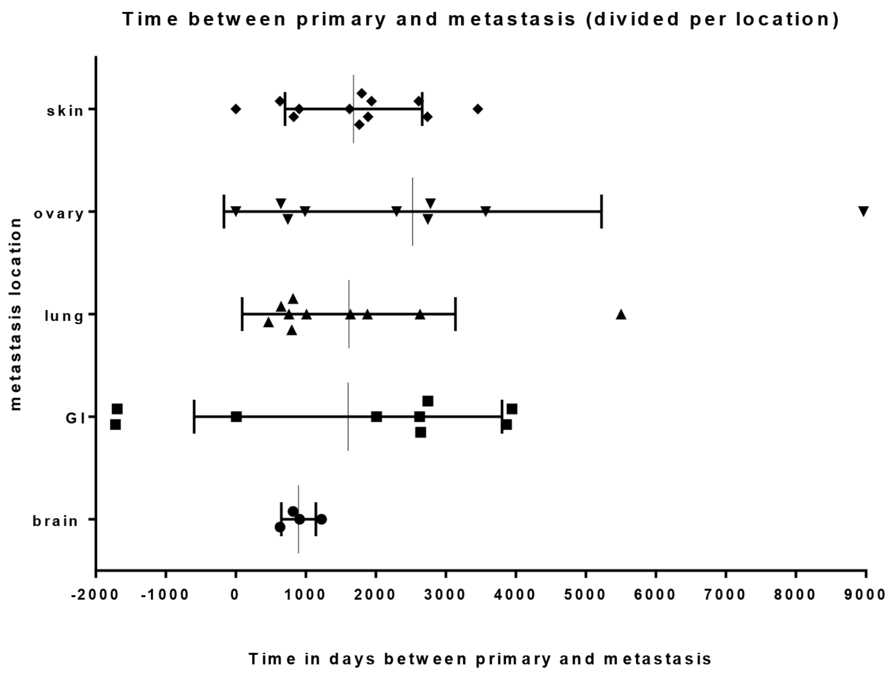

Supplementary Figure S5: Time in days between paired primary tumors and metastases per location for cohort 1. No significant differences were seen between locations.

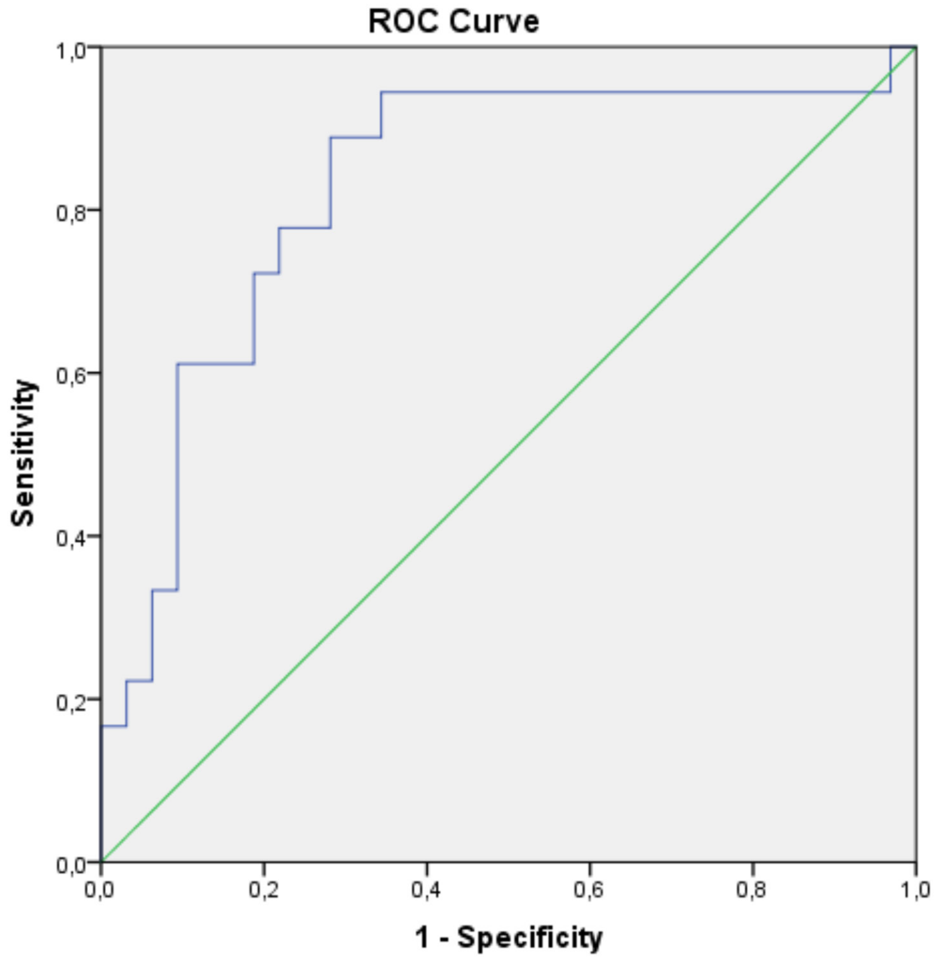

Supplementary Figure S6: ROC curve of hsa-miR-106b-5p qPCR expression in primary tumors that disseminated to lung vs. primary tumors that not disseminate to lung (cohort 1 and 2 together).

Supplementary Table S1: Characteristics of normal tissue included for determination of background microRNA levels using qPCR

Normal tissue	Subgroup	Age
Breast (n=4)	Range	22-55
	Mean	43
Lung (n=4)	Range	17-59
	Mean	44
Skin (n=4)	Range	42-65
	Mean	50
Ovary (n=4)	Range	52-83
	Mean	70
GI (n=4)	Range	25-79
	Mean	63

Supplementary Table S2: Location of metastases per patient in both cohorts.

See Supplementary File 1

Supplementary Table S3: Analyses on microarray data of cohort 1. 1) Threshold filtered data. 2) primary tumors versus metastases (unpaired). 3) primary tumors versus metastases (paired per location of metastasis). 4) site of metastasis predictive miRs in primary tumor. 5) site of metastasis predicting miRs in the metastasis. 6) other analyses.

See Supplementary File 2

Supplementary Table S4: Correlations of Threshold filtered data from the Exiqon microarray versus RQ data from SYBR Green qPCR assays of the most deregulated miRs in cohort 1

miRs	Sig. (2-tailed)	Spearman's rho	Analysis group
miR-106b-5p	0.017	0.294	Predictive in primary tumors for lung and GI metastases
miR-125b-5p	0.008	0.435	Upregulated in ovarian metastases versus primary tumors (paired)
miR-1273g-3p	0.020	0.285	Predictive in primary tumors for ovarian metastases
miR-143-3p	0.050	0.328	Upregulated in ovarian metastases versus primary tumors (paired)
miR-16-5p	0.204*	0.217	
miR-200a-3p	0.008	0.445	Significantly upregulated in metastases versus primary tumors (unpaired)
miR-29b-3p	0.017	0.400	Significantly upregulated in metastases versus primary tumors (unpaired)
miR-3182	0.000	0.720	Significantly upregulated in brain metastases versus non brain metastases
miR-451a	0.000	0.777	Significantly upregulated in metastases versus primary tumors (unpaired)
miR-7-5p	0.015	0.300	Predictive in primary tumors for skin metastases

* non-significant correlations

Supplementary Table S5: 38 oncogenic and tumor suppressive miRs that play a role in the metastatic cascade and are expressed in primary breast tumors

See Supplementary File 3

Supplementary Table S6: hsa-miR-106b-5p, hsa-miR-1273g-3p and hsa-miR-7-5p were inserted in miRTarBase to find experimentally validated microRNA-target interactions. The mRNA targets with strong validation evidence (obtained by reporter assay, western blot or qPCR) are listed. These target genes were subsequently imported in ToppGene Suite to show enriched pathways.

See Supplementary File 4
