# Supplementary material for: Unravelling site-specific breast cancer metastasis: a microRNA expression profiling study
Source: Oncotarget. 2016 Nov 25;8(2):3111–23. doi: 10.18632/oncotarget.13623 (PMC5356868; doi:10.18632/oncotarget.13623)
Supplement: Supplementary file 2 [file oncotarget-08-3111-s002.docx]

**Supplementary Table S2:** Location of metastases per patient in both cohorts.

| **Cohort** | **Patient #** | **Metastases included**  **in this study** | **Metastases to other organs**  **(no tumor material available)** |
| --- | --- | --- | --- |
| 1st | 1 | skin-ovary | bone |
| 1st | 2 | skin-ovary | lung, bone |
| 1st | 3 | skin-ovary | liver, bone, brain |
| 1st | 4 | skin-lung | bone, brain, liver |
| 1st | 5 | skin-lung | bone, liver |
| 1st | 6 | skin-lung |  |
| 1st | 8 | skin-skin |  |
| 1st | 9 | skin-skin | bone |
| 1st | 10 | skin-skin | bone |
| 1st | 11 | lung-ovary |  |
| 1st | 12 | lung-ovary | liver |
| 1st | 13 | lung-ovary | skin |
| 1st | 14 | lung-brain |  |
| 1st | 15 | lung-brain |  |
| 1st | 16 | lung-brain | bone |
| 1st | 17 | lung-brain |  |
| 1st | 18 | ovary-GI |  |
| 1st | 19 | ovary-GI | bone |
| 1st | 20 | ovary-GI |  |
| 1st | 21 | ovary-GI | bone, skin, liver, uterus |
| 1st | 23 | GI-GI | bone, liver |
| 1st | 24 | GI-GI | skin, liver, bone |
| 1st | 25 | GI-GI |  |
| 2nd | 101 | brain | lung |
| 2nd | 102 | brain |  |
| 2nd | 103 | brain | lung |
| 2nd | 104 | brain |  |
| 2nd | 105 | brain |  |
| 2nd | 106 | brain | bone |
| 2nd | 107 | brain |  |
| 2nd | 108 | lung | bone |
| 2nd | 109 | lung | bone |
| 2nd | 110 | lung |  |
| 2nd | 111 | lung |  |
| 2nd | 112 | lung |  |
| 2nd | 113 | lung |  |
| 2nd | 114 | skin | bone, liver, lung |
| 2nd | 115 | skin-GI |  |
| 2nd | 116 | skin | brain |
| 2nd | 117 | skin | bone |
| 2nd | 118 | skin |  |
| 2nd | 120 | ovary | bone, liver |
| 2nd | 121 | ovary | brain |
| 2nd | 122 | ovary | liver |
| 2nd | 123 | ovary | bone, uterus, GI |
| 2nd | 124 | ovary |  |
| 2nd | 125 | ovary |  |
| 2nd | 128 | GI | liver |
| 2nd | 129 | GI |  |
| 2nd | 130 | GI |  |
| 2nd | 131 | GI |  |
| 2nd | 132 | GI | ovary |
