# Supplementary material for: Unravelling site-specific breast cancer metastasis: a microRNA expression profiling study
Source: Oncotarget. 2016 Nov 25;8(2):3111–23. doi: 10.18632/oncotarget.13623 (PMC5356868; doi:10.18632/oncotarget.13623)
Supplement: Supplementary file 4 [file oncotarget-08-3111-s004.docx]

**Supplementary Table S5.** 38 oncogenic and tumor suppressive miRs that play a role in the metastatic cascade and are expressed in primary breast tumors.

| **miR** | **Type** | **Role** | **Ref** | **In Figure 4** |
| --- | --- | --- | --- | --- |
| 7 | Tumor suppressive | EMT, anoikis | [1-4] | Yes |
| 9 | Oncogenic | EMT, invasion, angiogenesis, metastasis | [5] | No* |
| 10b | Oncogenic | Migration, invasion, metastasis | [6-12] | Yes |
| 17/20 | Tumor suppressive | Proliferation | [13, 14] | No* |
| 17/92 | Tumor suppressive and oncogenic | NK antitumoral activity, metastasis, invasion | [15-17] | No$* |
| 21 | Oncogenic | Invasion, metastasis, migration, EMT, apoptosis | [18-28] | Yes |
| 22 | Tumor suppressive | Growth, senescence, metastasis, invasion | [29-31] | Yes |
| 23 | Oncogenic | Invasion, angiogenesis | [32] | Yes |
| 24 | Oncogenic | Metastasis, invasion | [33] | Yes |
| 29a/b | Oncogenic | EMT, metastasis, angiogenesis, invasion | [34, 35] | Yes |
| 30 | Tumor suppressive | Apoptosis, tumorigenesis, metastasis | [36, 37] | No* |
| 31 | Tumor suppressive | Invasion, metastasis, progression, intra- and extravasation | [38, 39] | No* |
| 34a/b/c | Tumor suppressive | Metastasis, invasion | [40-42] | No* |
| 93 | Oncogenic | Metastasis, angiogenesis, invasion | [43] | Yes |
| 103/107 | Oncogenic | EMT, migration, invasion, metastasis | [44] | Yes |
| 124 | Tumor suppressive | Metastasis, EMT, invasion | [45] | No* |
| 125b | Tumor suppressive and oncogenic | Proliferation, differentiation, migration, invasion | [46-49] | No$ |
| 126 | Tumor suppressive | Metastasis, angiogenesis, tumorigenesis, proliferation, cell adhesion | [50-56] | No* |
| 127/197/  222/223 | Tumor suppressive | Quiescence, proliferation | [57] | No* |
| 132 | Oncogenic | Neovascularization | [58] | No* |
| 145 | Tumor suppressive | Apoptosis, cell motility, invasion, metastasis, EMT, angiogenesis | [59-62] | No* |
| 146a/b | Tumor suppressive | Survival, metastasis, invasion, migration | [63-65] | Yes |
| 150 | Oncogenic | EMT | [66] | Yes |
| 155 | Tumor suppressive | Growth, proliferation, survival, EMT, migration, invasion | [67-71] | Yes |
| 182 | Oncogenic | EMT | [72, 73] | Yes |
| 193b | Tumor suppressive | Migration, invasion, metastasis | [74] | No* |
| 194 | Tumor suppressive | Invasion, EMT | [75] | Yes |
| 199 | Oncogenic | Angiogenesis | [76] | Yes |
| 200  family | Tumor suppressive and oncogenic | Growth, metastasis, EMT, MET, migration, invasion, proliferation, apoptosis | [77-92] | No$ |
| 205 | Tumor suppressive | Proliferation, invasion, EMT, metastasis | [84, 93-96] | Yes |
| 206 | Tumor suppressive | Migration, invasion, metastasis, apoptosis | [97-102] | No* |
| 221/222 | Oncogenic | EMT, invasion | [99, 103-106] | Yes |
| 335 | Tumor suppressive | Metastasis, migration, invasion | [10, 51, 107] | Yes |
| 373/520c | Oncogenic | Migration, invasion, metastasis | [10, 108, 109] | No* |
| 448 | Tumor suppressive | EMT, metastasis | [110] | No* |
| 661 | Tumor suppressive | Cell motility, invasion, EMT | [111, 112] | No* |
| 1908 | Oncogenic | Angiogenesis | [76] | Yes |
| Let-7  family | Tumor suppressive | Metastasis, invasion | [113-115] | Yes |

* not present in all tested samples

$ both tumor suppressive and oncogenic properties

**Reference list**

1. Foekens JA, Sieuwerts AM, Smid M, Look MP, de Weerd V, Boersma AW, Klijn JG, Wiemer EA, Martens JW. Four miRNAs associated with aggressiveness of lymph node-negative, estrogen receptor-positive human breast cancer. Proceedings of the National Academy of Sciences of the United States of America. 2008; 105: 13021-13026.

2. Webster RJ, Giles KM, Price KJ, Zhang PM, Mattick JS, Leedman PJ. Regulation of epidermal growth factor receptor signaling in human cancer cells by microRNA-7. The Journal of biological chemistry. 2009; 284: 5731-5741.

3. Reddy SD, Ohshiro K, Rayala SK, Kumar R. MicroRNA-7, a homeobox D10 target, inhibits p21-activated kinase 1 and regulates its functions. Cancer research. 2008; 68: 8195-8200.

4. Kong X, Li G, Yuan Y, He Y, Wu X, Zhang W, Wu Z, Chen T, Wu W, Lobie PE, Zhu T. MicroRNA-7 inhibits epithelial-to-mesenchymal transition and metastasis of breast cancer cells via targeting FAK expression. PloS one. 2012; 7: e41523.

5. Ma L, Young J, Prabhala H, Pan E, Mestdagh P, Muth D, Teruya-Feldstein J, Reinhardt F, Onder TT, Valastyan S, Westermann F, Speleman F, Vandesompele J, et al. miR-9, a MYC/MYCN-activated microRNA, regulates E-cadherin and cancer metastasis. Nature cell biology. 2010; 12: 247-256.

6. Tang J, Ahmad A, Sarkar FH. The role of microRNAs in breast cancer migration, invasion and metastasis. International journal of molecular sciences. 2012; 13: 13414-13437.

7. Ma L, Teruya-Feldstein J, Weinberg RA. Tumour invasion and metastasis initiated by microRNA-10b in breast cancer. Nature. 2007; 449: 682-688.

8. Gabriely G, Teplyuk NM, Krichevsky AM. Context effect: microRNA-10b in cancer cell proliferation, spread and death. Autophagy. 2011; 7: 1384-1386.

9. Bourguignon LY, Wong G, Earle C, Krueger K, Spevak CC. Hyaluronan-CD44 interaction promotes c-Src-mediated twist signaling, microRNA-10b expression, and RhoA/RhoC up-regulation, leading to Rho-kinase-associated cytoskeleton activation and breast tumor cell invasion. The Journal of biological chemistry. 2010; 285: 36721-36735.

10. Edmonds MD, Hurst DR, Vaidya KS, Stafford LJ, Chen D, Welch DR. Breast cancer metastasis suppressor 1 coordinately regulates metastasis-associated microRNA expression. International journal of cancer. 2009; 125: 1778-1785.

11. Moriarty CH, Pursell B, Mercurio AM. miR-10b targets Tiam1: implications for Rac activation and carcinoma migration. The Journal of biological chemistry. 2010; 285: 20541-20546.

12. Ma L, Reinhardt F, Pan E, Soutschek J, Bhat B, Marcusson EG, Teruya-Feldstein J, Bell GW, Weinberg RA. Therapeutic silencing of miR-10b inhibits metastasis in a mouse mammary tumor model. Nature biotechnology. 2010; 28: 341-347.

13. Yu Z, Wang C, Wang M, Li Z, Casimiro MC, Liu M, Wu K, Whittle J, Ju X, Hyslop T, McCue P, Pestell RG. A cyclin D1/microRNA 17/20 regulatory feedback loop in control of breast cancer cell proliferation. The Journal of cell biology. 2008; 182: 509-517.

14. Yu Z, Willmarth NE, Zhou J, Katiyar S, Wang M, Liu Y, McCue PA, Quong AA, Lisanti MP, Pestell RG. microRNA 17/20 inhibits cellular invasion and tumor metastasis in breast cancer by heterotypic signaling. Proceedings of the National Academy of Sciences of the United States of America. 2010; 107: 8231-8236.

15. Jiang H, Wang P, Li X, Wang Q, Deng ZB, Zhuang X, Mu J, Zhang L, Wang B, Yan J, Miller D, Zhang HG. Restoration of miR17/20a in solid tumor cells enhances the natural killer cell antitumor activity by targeting Mekk2. Cancer immunology research. 2014; 2: 789-799.

16. Fan M, Sethuraman A, Brown M, Sun W, Pfeffer LM. Systematic analysis of metastasis-associated genes identifies miR-17-5p as a metastatic suppressor of basal-like breast cancer. Breast cancer research and treatment. 2014; 146: 487-502.

17. Liu S, Goldstein RH, Scepansky EM, Rosenblatt M. Inhibition of rho-associated kinase signaling prevents breast cancer metastasis to human bone. Cancer research. 2009; 69: 8742-8751.

18. Huang TH, Wu F, Loeb GB, Hsu R, Heidersbach A, Brincat A, Horiuchi D, Lebbink RJ, Mo YY, Goga A, McManus MT. Up-regulation of miR-21 by HER2/neu signaling promotes cell invasion. The Journal of biological chemistry. 2009; 284: 18515-18524.

19. Han M, Wang Y, Liu M, Bi X, Bao J, Zeng N, Zhu Z, Mo Z, Wu C, Chen X. MiR-21 regulates epithelial-mesenchymal transition phenotype and hypoxia-inducible factor-1alpha expression in third-sphere forming breast cancer stem cell-like cells. Cancer science. 2012; 103: 1058-1064.

20. Lu Z, Liu M, Stribinskis V, Klinge CM, Ramos KS, Colburn NH, Li Y. MicroRNA-21 promotes cell transformation by targeting the programmed cell death 4 gene. Oncogene. 2008; 27: 4373-4379.

21. Qi L, Bart J, Tan LP, Platteel I, Sluis T, Huitema S, Harms G, Fu L, Hollema H, Berg A. Expression of miR-21 and its targets (PTEN, PDCD4, TM1) in flat epithelial atypia of the breast in relation to ductal carcinoma in situ and invasive carcinoma. BMC cancer. 2009; 9: 163-2407-9-163.

22. Song B, Wang C, Liu J, Wang X, Lv L, Wei L, Xie L, Zheng Y, Song X. MicroRNA-21 regulates breast cancer invasion partly by targeting tissue inhibitor of metalloproteinase 3 expression. Journal of experimental & clinical cancer research : CR. 2010; 29: 29-9966-29-29.

23. Baffa R, Fassan M, Volinia S, O'Hara B, Liu CG, Palazzo JP, Gardiman M, Rugge M, Gomella LG, Croce CM, Rosenberg A. MicroRNA expression profiling of human metastatic cancers identifies cancer gene targets. The Journal of pathology. 2009; 219: 214-221.

24. Connolly EC, Van Doorslaer K, Rogler LE, Rogler CE. Overexpression of miR-21 promotes an in vitro metastatic phenotype by targeting the tumor suppressor RHOB. Molecular cancer research : MCR. 2010; 8: 691-700.

25. Du J, Yang S, An D, Hu F, Yuan W, Zhai C, Zhu T. BMP-6 inhibits microRNA-21 expression in breast cancer through repressing deltaEF1 and AP-1. Cell research. 2009; 19: 487-496.

26. Yan LX, Huang XF, Shao Q, Huang MY, Deng L, Wu QL, Zeng YX, Shao JY. MicroRNA miR-21 overexpression in human breast cancer is associated with advanced clinical stage, lymph node metastasis and patient poor prognosis. RNA (New York, N.Y.). 2008; 14: 2348-2360.

27. Zhu S, Si ML, Wu H, Mo YY. MicroRNA-21 targets the tumor suppressor gene tropomyosin 1 (TPM1). The Journal of biological chemistry. 2007; 282: 14328-14336.

28. Zhu S, Wu H, Wu F, Nie D, Sheng S, Mo YY. MicroRNA-21 targets tumor suppressor genes in invasion and metastasis. Cell research. 2008; 18: 350-359.

29. Pandey DP, Picard D. miR-22 inhibits estrogen signaling by directly targeting the estrogen receptor alpha mRNA. Molecular and cellular biology. 2009; 29: 3783-3790.

30. Xu D, Takeshita F, Hino Y, Fukunaga S, Kudo Y, Tamaki A, Matsunaga J, Takahashi RU, Takata T, Shimamoto A, Ochiya T, Tahara H. miR-22 represses cancer progression by inducing cellular senescence. The Journal of cell biology. 2011; 193: 409-424.

31. Patel JB, Appaiah HN, Burnett RM, Bhat-Nakshatri P, Wang G, Mehta R, Badve S, Thomson MJ, Hammond S, Steeg P, Liu Y, Nakshatri H. Control of EVI-1 oncogene expression in metastatic breast cancer cells through microRNA miR-22. Oncogene. 2011; 30: 1290-1301.

32. Zhang H, Hao Y, Yang J, Zhou Y, Li J, Yin S, Sun C, Ma M, Huang Y, Xi JJ. Genome-wide functional screening of miR-23b as a pleiotropic modulator suppressing cancer metastasis. Nature communications. 2011; 2: 554.

33. Du WW, Fang L, Li M, Yang X, Liang Y, Peng C, Qian W, O'Malley YQ, Askeland RW, Sugg SL, Qian J, Lin J, Jiang Z, et al. MicroRNA miR-24 enhances tumor invasion and metastasis by targeting PTPN9 and PTPRF to promote EGF signaling. Journal of cell science. 2013; 126: 1440-1453.

34. Gebeshuber CA, Zatloukal K, Martinez J. miR-29a suppresses tristetraprolin, which is a regulator of epithelial polarity and metastasis. EMBO reports. 2009; 10: 400-405.

35. Chou J, Lin JH, Brenot A, Kim JW, Provot S, Werb Z. GATA3 suppresses metastasis and modulates the tumour microenvironment by regulating microRNA-29b expression. Nature cell biology. 2013; 15: 201-213.

36. Yu F, Deng H, Yao H, Liu Q, Su F, Song E. Mir-30 reduction maintains self-renewal and inhibits apoptosis in breast tumor-initiating cells. Oncogene. 2010; 29: 4194-4204.

37. Wu F, Zhu S, Ding Y, Beck WT, Mo YY. MicroRNA-mediated regulation of Ubc9 expression in cancer cells. Clinical cancer research : an official journal of the American Association for Cancer Research. 2009; 15: 1550-1557.

38. Augoff K, McCue B, Plow EF, Sossey-Alaoui K. miR-31 and its host gene lncRNA LOC554202 are regulated by promoter hypermethylation in triple-negative breast cancer. Molecular cancer. 2012; 11: 5-4598-11-5.

39. Sossey-Alaoui K, Downs-Kelly E, Das M, Izem L, Tubbs R, Plow EF. WAVE3, an actin remodeling protein, is regulated by the metastasis suppressor microRNA, miR-31, during the invasion-metastasis cascade. International journal of cancer. 2011; 129: 1331-1343.

40. Lujambio A, Calin GA, Villanueva A, Ropero S, Sanchez-Cespedes M, Blanco D, Montuenga LM, Rossi S, Nicoloso MS, Faller WJ, Gallagher WM, Eccles SA, Croce CM, et al. A microRNA DNA methylation signature for human cancer metastasis. Proceedings of the National Academy of Sciences of the United States of America. 2008; 105: 13556-13561.

41. Ahn YH, Gibbons DL, Chakravarti D, Creighton CJ, Rizvi ZH, Adams HP, Pertsemlidis A, Gregory PA, Wright JA, Goodall GJ, Flores ER, Kurie JM. ZEB1 drives prometastatic actin cytoskeletal remodeling by downregulating miR-34a expression. The Journal of clinical investigation. 2012; 122: 3170-3183.

42. Yang S, Li Y, Gao J, Zhang T, Li S, Luo A, Chen H, Ding F, Wang X, Liu Z. MicroRNA-34 suppresses breast cancer invasion and metastasis by directly targeting Fra-1. Oncogene. 2013; 32: 4294-4303.

43. Fang L, Du WW, Yang W, Rutnam ZJ, Peng C, Li H, O'Malley YQ, Askeland RW, Sugg S, Liu M, Mehta T, Deng Z, Yang BB. MiR-93 enhances angiogenesis and metastasis by targeting LATS2. Cell cycle (Georgetown, Tex.). 2012; 11: 4352-4365.

44. Martello G, Rosato A, Ferrari F, Manfrin A, Cordenonsi M, Dupont S, Enzo E, Guzzardo V, Rondina M, Spruce T, Parenti AR, Daidone MG, Bicciato S, et al. A MicroRNA targeting dicer for metastasis control. Cell. 2010; 141: 1195-1207.

45. Liang YJ, Wang QY, Zhou CX, Yin QQ, He M, Yu XT, Cao DX, Chen GQ, He JR, Zhao Q. MiR-124 targets Slug to regulate epithelial-mesenchymal transition and metastasis of breast cancer. Carcinogenesis. 2013; 34: 713-722.

46. Feliciano A, Castellvi J, Artero-Castro A, Leal JA, Romagosa C, Hernandez-Losa J, Peg V, Fabra A, Vidal F, Kondoh H, Ramon Y Cajal S, Lleonart ME. miR-125b acts as a tumor suppressor in breast tumorigenesis via its novel direct targets ENPEP, CK2-alpha, CCNJ, and MEGF9. PloS one. 2013; 8: e76247.

47. Scott GK, Goga A, Bhaumik D, Berger CE, Sullivan CS, Benz CC. Coordinate suppression of ERBB2 and ERBB3 by enforced expression of micro-RNA miR-125a or miR-125b. The Journal of biological chemistry. 2007; 282: 1479-1486.

48. Ferracin M, Bassi C, Pedriali M, Pagotto S, D'Abundo L, Zagatti B, Corra F, Musa G, Callegari E, Lupini L, Volpato S, Querzoli P, Negrini M. miR-125b targets erythropoietin and its receptor and their expression correlates with metastatic potential and ERBB2/HER2 expression. Molecular cancer. 2013; 12: 130-4598-12-130.

49. Tang F, Zhang R, He Y, Zou M, Guo L, Xi T. MicroRNA-125b induces metastasis by targeting STARD13 in MCF-7 and MDA-MB-231 breast cancer cells. PloS one. 2012; 7: e35435.

50. Png KJ, Halberg N, Yoshida M, Tavazoie SF. A microRNA regulon that mediates endothelial recruitment and metastasis by cancer cells. Nature. 2011; 481: 190-194.

51. Tavazoie SF, Alarcon C, Oskarsson T, Padua D, Wang Q, Bos PD, Gerald WL, Massague J. Endogenous human microRNAs that suppress breast cancer metastasis. Nature. 2008; 451: 147-152.

52. Fish JE, Santoro MM, Morton SU, Yu S, Yeh RF, Wythe JD, Ivey KN, Bruneau BG, Stainier DY, Srivastava D. miR-126 regulates angiogenic signaling and vascular integrity. Developmental cell. 2008; 15: 272-284.

53. Harris TA, Yamakuchi M, Ferlito M, Mendell JT, Lowenstein CJ. MicroRNA-126 regulates endothelial expression of vascular cell adhesion molecule 1. Proceedings of the National Academy of Sciences of the United States of America. 2008; 105: 1516-1521.

54. Sempere LF, Preis M, Yezefski T, Ouyang H, Suriawinata AA, Silahtaroglu A, Conejo-Garcia JR, Kauppinen S, Wells W, Korc M. Fluorescence-based codetection with protein markers reveals distinct cellular compartments for altered MicroRNA expression in solid tumors. Clinical cancer research : an official journal of the American Association for Cancer Research. 2010; 16: 4246-4255.

55. Li X, Shen Y, Ichikawa H, Antes T, Goldberg GS. Regulation of miRNA expression by Src and contact normalization: effects on nonanchored cell growth and migration. Oncogene. 2009; 28: 4272-4283.

56. Zhang Y, Yang P, Sun T, Li D, Xu X, Rui Y, Li C, Chong M, Ibrahim T, Mercatali L, Amadori D, Lu X, Xie D, et al. miR-126 and miR-126* repress recruitment of mesenchymal stem cells and inflammatory monocytes to inhibit breast cancer metastasis. Nature cell biology. 2013; 15: 284-294.

57. Lim PK, Bliss SA, Patel SA, Taborga M, Dave MA, Gregory LA, Greco SJ, Bryan M, Patel PS, Rameshwar P. Gap junction-mediated import of microRNA from bone marrow stromal cells can elicit cell cycle quiescence in breast cancer cells. Cancer research. 2011; 71: 1550-1560.

58. Anand S, Majeti BK, Acevedo LM, Murphy EA, Mukthavaram R, Scheppke L, Huang M, Shields DJ, Lindquist JN, Lapinski PE, King PD, Weis SM, Cheresh DA. MicroRNA-132-mediated loss of p120RasGAP activates the endothelium to facilitate pathological angiogenesis. Nature medicine. 2010; 16: 909-914.

59. Sachdeva M, Zhu S, Wu F, Wu H, Walia V, Kumar S, Elble R, Watabe K, Mo YY. p53 represses c-Myc through induction of the tumor suppressor miR-145. Proceedings of the National Academy of Sciences of the United States of America. 2009; 106: 3207-3212.

60. Sachdeva M, Mo YY. MicroRNA-145 suppresses cell invasion and metastasis by directly targeting mucin 1. Cancer research. 2010; 70: 378-387.

61. Spizzo R, Nicoloso MS, Lupini L, Lu Y, Fogarty J, Rossi S, Zagatti B, Fabbri M, Veronese A, Liu X, Davuluri R, Croce CM, Mills G, et al. miR-145 participates with TP53 in a death-promoting regulatory loop and targets estrogen receptor-alpha in human breast cancer cells. Cell death and differentiation. 2010; 17: 246-254.

62. Gotte M, Mohr C, Koo CY, Stock C, Vaske AK, Viola M, Ibrahim SA, Peddibhotla S, Teng YH, Low JY, Ebnet K, Kiesel L, Yip GW. miR-145-dependent targeting of junctional adhesion molecule A and modulation of fascin expression are associated with reduced breast cancer cell motility and invasiveness. Oncogene. 2010; 29: 6569-6580.

63. Xiang M, Birkbak NJ, Vafaizadeh V, Walker SR, Yeh JE, Liu S, Kroll Y, Boldin M, Taganov K, Groner B, Richardson AL, Frank DA. STAT3 induction of miR-146b forms a feedback loop to inhibit the NF-kappaB to IL-6 signaling axis and STAT3-driven cancer phenotypes. Science signaling. 2014; 7: ra11.

64. Bhaumik D, Scott GK, Schokrpur S, Patil CK, Campisi J, Benz CC. Expression of microRNA-146 suppresses NF-kappaB activity with reduction of metastatic potential in breast cancer cells. Oncogene. 2008; 27: 5643-5647.

65. Hurst DR, Edmonds MD, Scott GK, Benz CC, Vaidya KS, Welch DR. Breast cancer metastasis suppressor 1 up-regulates miR-146, which suppresses breast cancer metastasis. Cancer research. 2009; 69: 1279-1283.

66. Yokobori T, Suzuki S, Tanaka N, Inose T, Sohda M, Sano A, Sakai M, Nakajima M, Miyazaki T, Kato H, Kuwano H. MiR-150 is associated with poor prognosis in esophageal squamous cell carcinoma via targeting the EMT inducer ZEB1. Cancer science. 2013; 104: 48-54.

67. Jiang S, Zhang HW, Lu MH, He XH, Li Y, Gu H, Liu MF, Wang ED. MicroRNA-155 functions as an OncomiR in breast cancer by targeting the suppressor of cytokine signaling 1 gene. Cancer research. 2010; 70: 3119-3127.

68. Zhang CM, Zhao J, Deng HY. MiR-155 promotes proliferation of human breast cancer MCF-7 cells through targeting tumor protein 53-induced nuclear protein 1. Journal of Biomedical Science. 2013; 20: 79-0127-20-79.

69. Kong W, Yang H, He L, Zhao JJ, Coppola D, Dalton WS, Cheng JQ. MicroRNA-155 is regulated by the transforming growth factor beta/Smad pathway and contributes to epithelial cell plasticity by targeting RhoA. Molecular and cellular biology. 2008; 28: 6773-6784.

70. Kong W, He L, Coppola M, Guo J, Esposito NN, Coppola D, Cheng JQ. MicroRNA-155 regulates cell survival, growth, and chemosensitivity by targeting FOXO3a in breast cancer. The Journal of biological chemistry. 2010; 285: 17869-17879.

71. Liang Z, Wu H, Reddy S, Zhu A, Wang S, Blevins D, Yoon Y, Zhang Y, Shim H. Blockade of invasion and metastasis of breast cancer cells via targeting CXCR4 with an artificial microRNA. Biochemical and biophysical research communications. 2007; 363: 542-546.

72. Chiang CH, Chu PY, Hou MF, Hung WC. MiR-182 promotes proliferation and invasion and elevates the HIF-1alpha-VEGF-A axis in breast cancer cells by targeting FBXW7. American journal of cancer research. 2016; 6: 1785-1798.

73. Fite K, Gomez-Cambronero J. Down-regulation of MicroRNAs (MiRs) 203, 887, 3619 and 182 Prevents Vimentin-triggered, Phospholipase D (PLD)-mediated Cancer Cell Invasion. The Journal of biological chemistry. 2016; 291: 719-730.

74. Li XF, Yan PJ, Shao ZM. Downregulation of miR-193b contributes to enhance urokinase-type plasminogen activator (uPA) expression and tumor progression and invasion in human breast cancer. Oncogene. 2009; 28: 3937-3948.

75. Meng Z, Fu X, Chen X, Zeng S, Tian Y, Jove R, Xu R, Huang W. miR-194 is a marker of hepatic epithelial cells and suppresses metastasis of liver cancer cells in mice. Hepatology (Baltimore, Md.). 2010; 52: 2148-2157.

76. Pencheva N, Tran H, Buss C, Huh D, Drobnjak M, Busam K, Tavazoie SF. Convergent multi-miRNA targeting of ApoE drives LRP1/LRP8-dependent melanoma metastasis and angiogenesis. Cell. 2012; 151: 1068-1082.

77. Zhang HF, Xu LY, Li EM. A family of pleiotropically acting microRNAs in cancer progression, miR-200: potential cancer therapeutic targets. Current pharmaceutical design. 2014; 20: 1896-1903.

78. Castilla MA, Diaz-Martin J, Sarrio D, Romero-Perez L, Lopez-Garcia MA, Vieites B, Biscuola M, Ramiro-Fuentes S, Isacke CM, Palacios J. MicroRNA-200 family modulation in distinct breast cancer phenotypes. PloS one. 2012; 7: e47709.

79. Roy SS, Gonugunta VK, Bandyopadhyay A, Rao MK, Goodall GJ, Sun LZ, Tekmal RR, Vadlamudi RK. Significance of PELP1/HDAC2/miR-200 regulatory network in EMT and metastasis of breast cancer. Oncogene. 2014; 33: 3707-3716.

80. Dykxhoorn DM, Wu Y, Xie H, Yu F, Lal A, Petrocca F, Martinvalet D, Song E, Lim B, Lieberman J. miR-200 enhances mouse breast cancer cell colonization to form distant metastases. PloS one. 2009; 4: e7181.

81. Bracken CP, Gregory PA, Kolesnikoff N, Bert AG, Wang J, Shannon MF, Goodall GJ. A double-negative feedback loop between ZEB1-SIP1 and the microRNA-200 family regulates epithelial-mesenchymal transition. Cancer research. 2008; 68: 7846-7854.

82. Burk U, Schubert J, Wellner U, Schmalhofer O, Vincan E, Spaderna S, Brabletz T. A reciprocal repression between ZEB1 and members of the miR-200 family promotes EMT and invasion in cancer cells. EMBO reports. 2008; 9: 582-589.

83. Gibbons DL, Lin W, Creighton CJ, Rizvi ZH, Gregory PA, Goodall GJ, Thilaganathan N, Du L, Zhang Y, Pertsemlidis A, Kurie JM. Contextual extracellular cues promote tumor cell EMT and metastasis by regulating miR-200 family expression. Genes & development. 2009; 23: 2140-2151.

84. Gregory PA, Bert AG, Paterson EL, Barry SC, Tsykin A, Farshid G, Vadas MA, Khew-Goodall Y, Goodall GJ. The miR-200 family and miR-205 regulate epithelial to mesenchymal transition by targeting ZEB1 and SIP1. Nature cell biology. 2008; 10: 593-601.

85. Iliopoulos D, Polytarchou C, Hatziapostolou M, Kottakis F, Maroulakou IG, Struhl K, Tsichlis PN. MicroRNAs differentially regulated by Akt isoforms control EMT and stem cell renewal in cancer cells. Science signaling. 2009; 2: ra62.

86. Iliopoulos D, Lindahl-Allen M, Polytarchou C, Hirsch HA, Tsichlis PN, Struhl K. Loss of miR-200 inhibition of Suz12 leads to polycomb-mediated repression required for the formation and maintenance of cancer stem cells. Molecular cell. 2010; 39: 761-772.

87. Li S, Wang Q, Wang Y, Chen X, Wang Z. PLC-gamma1 and Rac1 coregulate EGF-induced cytoskeleton remodeling and cell migration. Molecular endocrinology (Baltimore, Md.). 2009; 23: 901-913.

88. Park SM, Gaur AB, Lengyel E, Peter ME. The miR-200 family determines the epithelial phenotype of cancer cells by targeting the E-cadherin repressors ZEB1 and ZEB2. Genes & development. 2008; 22: 894-907.

89. Schickel R, Park SM, Murmann AE, Peter ME. miR-200c regulates induction of apoptosis through CD95 by targeting FAP-1. Molecular cell. 2010; 38: 908-915.

90. Uhlmann S, Zhang JD, Schwager A, Mannsperger H, Riazalhosseini Y, Burmester S, Ward A, Korf U, Wiemann S, Sahin O. miR-200bc/429 cluster targets PLCgamma1 and differentially regulates proliferation and EGF-driven invasion than miR-200a/141 in breast cancer. Oncogene. 2010; 29: 4297-4306.

91. Korpal M, Ell BJ, Buffa FM, Ibrahim T, Blanco MA, Celia-Terrassa T, Mercatali L, Khan Z, Goodarzi H, Hua Y, Wei Y, Hu G, Garcia BA, et al. Direct targeting of Sec23a by miR-200s influences cancer cell secretome and promotes metastatic colonization. Nature medicine. 2011; 17: 1101-1108.

92. Yu SJ, Hu JY, Kuang XY, Luo JM, Hou YF, Di GH, Wu J, Shen ZZ, Song HY, Shao ZM. MicroRNA-200a promotes anoikis resistance and metastasis by targeting YAP1 in human breast cancer. Clinical cancer research : an official journal of the American Association for Cancer Research. 2013; 19: 1389-1399.

93. Liu J, Mao Q, Liu Y, Hao X, Zhang S, Zhang J. Analysis of miR-205 and miR-155 expression in the blood of breast cancer patients. Chinese journal of cancer research = Chung-kuo yen cheng yen chiu. 2013; 25: 46-54.

94. Elgamal OA, Park JK, Gusev Y, Azevedo-Pouly AC, Jiang J, Roopra A, Schmittgen TD. Tumor suppressive function of mir-205 in breast cancer is linked to HMGB3 regulation. PloS one. 2013; 8: e76402.

95. Iorio MV, Casalini P, Piovan C, Di Leva G, Merlo A, Triulzi T, Menard S, Croce CM, Tagliabue E. microRNA-205 regulates HER3 in human breast cancer. Cancer research. 2009; 69: 2195-2200.

96. Wu H, Zhu S, Mo YY. Suppression of cell growth and invasion by miR-205 in breast cancer. Cell research. 2009; 19: 439-448.

97. Li Y, Hong F, Yu Z. Decreased expression of microRNA-206 in breast cancer and its association with disease characteristics and patient survival. The Journal of international medical research. 2013; 41: 596-602.

98. Song G, Zhang Y, Wang L. MicroRNA-206 targets notch3, activates apoptosis, and inhibits tumor cell migration and focus formation. The Journal of biological chemistry. 2009; 284: 31921-31927.

99. Di Leva G, Gasparini P, Piovan C, Ngankeu A, Garofalo M, Taccioli C, Iorio MV, Li M, Volinia S, Alder H, Nakamura T, Nuovo G, Liu Y, et al. MicroRNA cluster 221-222 and estrogen receptor alpha interactions in breast cancer. Journal of the National Cancer Institute. 2010; 102: 706-721.

100. Leivonen SK, Makela R, Ostling P, Kohonen P, Haapa-Paananen S, Kleivi K, Enerly E, Aakula A, Hellstrom K, Sahlberg N, Kristensen VN, Borresen-Dale AL, Saviranta P, et al. Protein lysate microarray analysis to identify microRNAs regulating estrogen receptor signaling in breast cancer cell lines. Oncogene. 2009; 28: 3926-3936.

101. Adams BD, Cowee DM, White BA. The role of miR-206 in the epidermal growth factor (EGF) induced repression of estrogen receptor-alpha (ERalpha) signaling and a luminal phenotype in MCF-7 breast cancer cells. Molecular endocrinology (Baltimore, Md.). 2009; 23: 1215-1230.

102. Kondo N, Toyama T, Sugiura H, Fujii Y, Yamashita H. miR-206 Expression is down-regulated in estrogen receptor alpha-positive human breast cancer. Cancer research. 2008; 68: 5004-5008.

103. Stinson S, Lackner MR, Adai AT, Yu N, Kim HJ, O'Brien C, Spoerke J, Jhunjhunwala S, Boyd Z, Januario T, Newman RJ, Yue P, Bourgon R, et al. TRPS1 targeting by miR-221/222 promotes the epithelial-to-mesenchymal transition in breast cancer. Science signaling. 2011; 4: ra41.

104. Hwang MS, Yu N, Stinson SY, Yue P, Newman RJ, Allan BB, Dornan D. miR-221/222 targets adiponectin receptor 1 to promote the epithelial-to-mesenchymal transition in breast cancer. PloS one. 2013; 8: e66502.

105. Nassirpour R, Mehta PP, Baxi SM, Yin MJ. miR-221 promotes tumorigenesis in human triple negative breast cancer cells. PloS one. 2013; 8: e62170.

106. Pichiorri F, Palmieri D, De Luca L, Consiglio J, You J, Rocci A, Talabere T, Piovan C, Lagana A, Cascione L, Guan J, Gasparini P, Balatti V, et al. In vivo NCL targeting affects breast cancer aggressiveness through miRNA regulation. The Journal of experimental medicine. 2013; 210: 951-968.

107. Zhang S, Kim K, Jin UH, Pfent C, Cao H, Amendt B, Liu X, Wilson-Robles H, Safe S. Aryl hydrocarbon receptor agonists induce microRNA-335 expression and inhibit lung metastasis of estrogen receptor negative breast cancer cells. Molecular cancer therapeutics. 2012; 11: 108-118.

108. Huang Q, Gumireddy K, Schrier M, le Sage C, Nagel R, Nair S, Egan DA, Li A, Huang G, Klein-Szanto AJ, Gimotty PA, Katsaros D, Coukos G, et al. The microRNAs miR-373 and miR-520c promote tumour invasion and metastasis. Nature cell biology. 2008; 10: 202-210.

109. Yan GR, Xu SH, Tan ZL, Liu L, He QY. Global identification of miR-373-regulated genes in breast cancer by quantitative proteomics. Proteomics. 2011; 11: 912-920.

110. Li QQ, Chen ZQ, Cao XX, Xu JD, Xu JW, Chen YY, Wang WJ, Chen Q, Tang F, Liu XP, Xu ZD. Involvement of NF-kappaB/miR-448 regulatory feedback loop in chemotherapy-induced epithelial-mesenchymal transition of breast cancer cells. Cell death and differentiation. 2011; 18: 16-25.

111. Reddy SD, Pakala SB, Ohshiro K, Rayala SK, Kumar R. MicroRNA-661, a c/EBPalpha target, inhibits metastatic tumor antigen 1 and regulates its functions. Cancer research. 2009; 69: 5639-5642.

112. Vetter G, Saumet A, Moes M, Vallar L, Le Bechec A, Laurini C, Sabbah M, Arar K, Theillet C, Lecellier CH, Friederich E. miR-661 expression in SNAI1-induced epithelial to mesenchymal transition contributes to breast cancer cell invasion by targeting Nectin-1 and StarD10 messengers. Oncogene. 2010; 29: 4436-4448.

113. Yu F, Yao H, Zhu P, Zhang X, Pan Q, Gong C, Huang Y, Hu X, Su F, Lieberman J, Song E. Let-7 Regulates Self Renewal and Tumorigenicity of Breast Cancer Cells. Cell. 2007; 131: 1109-1123.

114. Qian P, Zuo Z, Wu Z, Meng X, Li G, Wu Z, Zhang W, Tan S, Pandey V, Yao Y, Wang P, Zhao L, Wang J, et al. Pivotal role of reduced let-7g expression in breast cancer invasion and metastasis. Cancer research. 2011; 71: 6463-6474.

115. Lamouille S, Subramanyam D, Blelloch R, Derynck R. Regulation of epithelial-mesenchymal and mesenchymal-epithelial transitions by microRNAs. Current opinion in cell biology. 2013; 25: 200-207.
